# Parametric Reconstruction and Pore-Scale Transport Analysis of Microporous Layers in PEM Fuel Cells

**DOI:** 10.3390/nano16090529

**Published:** 2026-04-27

**Authors:** Shengbo Sun, Lingquan Li, Hao Wang, Guogang Yang

**Affiliations:** Marine Engineering College, Dalian Maritime University, Dalian 116026, China; sunshengbo@dlmu.edu.cn (S.S.); li_0203@dlmu.edu.cn (L.L.)

**Keywords:** proton exchange membrane fuel cell, microporous layer, stochastic reconstruction, random walk, transport properties, structural optimization

## Abstract

The microporous layer (MPL) is a key functional component in proton exchange membrane fuel cells (PEMFCs), and clarifying the quantitative relationship between its microstructure and mass transport properties is essential for improving cell performance. In this study, a three-dimensional MPL model was developed using a stochastic reconstruction method, and, together with a random walk algorithm, was employed to systematically investigate the effects of porosity, carbon sphere radius, maximum overlap ratio, seed ratio, and polytetrafluoroethylene (PTFE) content on permeability, effective diffusivity, and tortuosity. The results reveal that increasing porosity reduces tortuosity from 1.7 to 1.3, while permeability and effective diffusivity increase by factors of approximately 6.5 and 1.8, respectively. As the carbon sphere radius increases, tortuosity decreases from 1.55 to 1.35, accompanied by an increase in permeability from 2 × 10^−16^ m^2^ to 20 × 10^−16^ m^2^. Moreover, increasing the PTFE content raises permeability from 5 × 10^−16^ m^2^ to 22.5 × 10^−16^ m^2^, corresponding to an enhancement by a factor of approximately 4.5. The high-accuracy fitting equations obtained from the simulation results provide theoretical guidance for the microstructural design and optimization of MPLs, which can enhance oxygen transport and water management, reduce mass transport losses, and thereby benefit high-power-density operation and the overall efficiency of PEM fuel cells.

## 1. Introduction

As a highly efficient energy conversion technology, the proton exchange membrane fuel cell (PEMFC) has garnered significant attention owing to its high efficiency and clean emission characteristics. Through electrochemical reactions, the PEMFC directly converts the chemical energy of hydrogen into electrical energy, achieving an energy conversion efficiency of over 60% with water as the primary reaction product, thereby demonstrating considerable potential for low-carbon energy utilization. Within the PEMFC, the microporous layer (MPL), serving as a functional layer between the catalyst layer and the gas diffusion layer, not only participates in regulating gas diffusion and permeation but also influences interfacial mass transport resistance and operational stability. Consequently, the MPL is recognized as a critical factor determining fuel cell performance and energy loss [1,2]. Particularly under high-power-density operating conditions, the permeability and oxygen diffusivity of the MPL play a critical role in determining reactant transport and distribution, thereby influencing mass transport polarization and ultimately affecting overall cell performance [3,4]. Therefore, a comprehensive investigation into the intrinsic relationship between the microstructure and effective transport parameters of the MPL, along with subsequent structural optimization, has emerged as a vital research direction for enhancing the performance and durability of PEMFCs.

In recent years, significant progress has been made in the frontier research of MPLs alongside the continuous development of fuel cell technology. Researchers have not only focused on the fundamental mass transport characteristics of MPLs but have also conducted in-depth investigations into structural optimization and multiphysics coupled simulations. Specifically, the reinforcement of MPLs using nanomaterials such as graphene and carbon nanotubes has been extensively explored, aiming to enhance their mechanical strength, electrical conductivity, and hydrophobicity. Previous studies have reported that such modifications can significantly improve fuel cell performance, with peak power density increases ranging from approximately 20% to over 50% under optimized conditions, along with enhanced mass transport and cell durability [5,6]. In addition, increasing attention has been directed toward multiscale modeling and numerical simulation. In particular, through high-resolution three-dimensional reconstruction techniques and stochastic reconstruction methods, the heterogeneity of MPL microstructures has been accurately captured, enabling the prediction of key transport properties including permeability, diffusivity, and tortuosity, where tortuosity describes the complexity of transport pathways within the porous medium and reflects the deviation of actual diffusion paths from straight lines, thereby influencing the effective diffusivity of reactants [7,8]. These cutting-edge studies have not only advanced the theoretical development of MPL structural optimization but also provided robust technical support for enhancing fuel cell performance by improving mass transport behavior, particularly oxygen diffusion and liquid water removal within the porous structure.

The transport behavior of the MPL is highly dependent on its microstructure and interfacial characteristics. Typical influencing factors include pore structure parameters such as porosity, pore size, pore connectivity, and PTFE content. These factors collectively determine the coupling relationship between liquid water removal and oxygen supply, thereby affecting mass transport losses (or concentration polarization) and performance degradation under various operating conditions. This issue has been explored from multiple perspectives in previous studies. Using porous transport modeling, Nouri-Khorasani et al. pointed out that in carbon-based MPLs with hierarchical pore structures, the pore structure, wettability, permeability, and contact quality with the catalyst layer collectively influence performance, and that optimizing material and interfacial properties contributes to enhanced overall fuel cell performance, with I-R compensated voltages improved by approximately 0.3 V at 2.0 A/cm^2^ [9]. Based on synchrotron X-ray imaging combined with in situ electrochemical testing, Lee et al. found that although multi-walled carbon nanotube MPLs may lead to higher liquid water saturation, oxygen transport was improved due to their larger pore size and higher porosity, resulting in enhanced performance, with power densities increasing by 6.7% and 94.1% at 2.0 A/cm^2^ and 2.5 A/cm^2^, respectively, compared to conventional MPLs [10]. On the other hand, pore-scale simulations have also revealed the sensitivity of transport properties to structural parameters. Ren et al. reconstructed catalyst layer and MPL nanostructures and calculated permeability using a three-dimensional lattice Boltzmann method, finding that permeability increased with increasing porosity and pore size [11]. Using nano-tomography and pore network modeling, Ma et al. demonstrated that cracks and pore connectivity significantly affect liquid water pathway formation and equivalent permeability [12]. By investigating the interface structure between the gas diffusion layer and the microporous layer using pore-scale models and lattice Boltzmann method simulations, Zhang et al. showed that high compression conditions significantly reduce gas diffusivity and liquid water permeability while enhancing anisotropic transport [13].

Although significant progress has been made in structural characterization, digital reconstruction, and pore-scale simulation, notable limitations remain in unified computational and parametric studies aimed at structural optimization. Firstly, many studies rely on three-dimensional reconstructed structures obtained from techniques such as computed tomography (CT) or FIB-SEM on single samples. Although these methods can capture realistic geometries, systematic scanning and optimization across multiple structural factors within a unified framework remain challenging [14]. A unified modeling approach that enables controllable generation, reproducible computation, and comparative analysis is still lacking for tunable structural parameters such as particle characteristic size, porosity, particle overlap degree, nucleation/seeding ratio, and PTFE content [15]. Secondly, systematic investigations at the pore scale regarding diffusion-related metrics such as oxygen effective diffusivity and tortuosity remain insufficient. Differences in assumptions regarding diffusion mechanisms, boundary condition settings, and parameter extraction methods across studies limit the comparability and transferability of the obtained results [16]. For instance, random walk methods have been applied to calculate tortuosity in catalyst layer structures, effectively capturing pore path tortuosity and diffusion performance; however, challenges persist in achieving unified, robust, and reproducible characterization across a multi-parameter space [17]. With respect to MPLs, systematic multi-parameter studies are still lacking on how hydrophobic components such as PTFE can be incorporated as explicit structural elements into three-dimensional digital reconstructions to quantify their effects on permeability, oxygen effective diffusivity, and tortuosity [18,19]. Similar deficiencies are also observed in studies on diffusion media such as carbon paper GDLs. Although some studies have employed random walk methods to calculate effective transport properties in randomly reconstructed carbon paper GDL structures and analyzed the influence of structural parameters on diffusion, the structural parameter systems in these studies differ from the typical particle packing and PTFE coupling mechanisms found in MPLs [20,21].

Based on the above considerations, a three-dimensional MPL microstructure model with tunable structural parameters was constructed using a stochastic reconstruction method. Combined with random walk algorithms and pore-scale transport calculations, the effects of porosity, carbon sphere radius, overlap ratio, seeding ratio, and PTFE content on the permeability, oxygen effective diffusivity, and tortuosity of the MPL were systematically evaluated. This study is expected to provide a theoretical basis for the quantitative structural design and optimization of MPLs, as these parameters critically govern gas diffusion kinetics and are expected to enhance fuel cell performance under high current densities, primarily by mitigating concentration polarization.

## 2. Methodology

### 2.1. Reconstruction of the MPL Microstructure

In this section, a stochastic reconstruction method was adopted to generate a three-dimensional structural model of the MPL. This method encompasses the generation procedures for carbon spheres and PTFE, enabling the random generation of the MPL by controlling parameters such as sphere radius, packing density, and overlap probability.

#### 2.1.1. Generation of Carbon

To construct the three-dimensional structural model of the MPL, carbon spheres, which represent the active phase of the MPL, were first generated. These carbon spheres were randomly distributed within the three-dimensional space, with a minimum distance maintained between spheres to avoid overlap, thereby simulating the spatial distribution of carbon particles. The generation of carbon spheres was performed using a random distribution approach, wherein the spheres were randomly placed in the three-dimensional space according to a specified radius range and distribution probability. The detailed procedure is as follows [15]:

(1) The radius of each carbon sphere was randomly distributed within a prescribed range between a minimum and maximum value. With the carbon sphere radius range defined, the radius *r_i_* of each generated carbon sphere was determined using Equation (1):

(1) $$r_{i}=r_{\min}+(r_{\max}-r_{\min})\times \mathrm{rand}(1)$$

where *r_i_* is the radius of the *i*-th carbon sphere, *r*_min_ and *r*_max_ are the minimum and maximum radius values, respectively, and rand(1) represents a uniformly distributed random number in the interval [0, 1], which determines the position of *r_i_* within the prescribed range.

(2) The sphere centers were randomly generated to identify non-overlapping regions within the space, and the carbon spheres were placed at these positions. The overlap condition for the carbon spheres was determined by calculating the distance between sphere centers and the sum of their radius. When the distance between two spheres was less than the sum of their radius, they were considered overlapping, and a new position was required to be re-selected.

(3) The volume of each sphere was calculated using Equation (2):

(2) $$V_{c}=\frac{4}{3}\pi r^{3}$$

(4) The number of carbon spheres $N_{c}$ and the total generated volume were controlled by setting a target porosity. As expressed in Equation (3), the volume target was achieved by iteratively adjusting the number of spheres until the target porosity was satisfied.

(3) $$\mathrm{Target}\;\mathrm{porosity}=\frac{N_{c}\times V_{c}}{\mathrm{volume}}$$

where *N_c_* is the number of carbon spheres, *V_c_* is the volume of a single carbon sphere, and volume represents the total volume of the MPL sample used for reconstruction.

#### 2.1.2. Generation of PTFE

The introduction of PTFE during MPL fabrication increases hydrophobicity, which is a critical factor in regulating water management performance. PTFE forms a hydrophobic phase by filling the voids between carbon spheres, thereby influencing water transport characteristics.

Within the MPL, PTFE serves dual roles as both a binder and a hydrophobic phase, and its spatial distribution directly affects liquid water migration pathways as well as gas channel connectivity. Based on two typical distribution patterns proposed in previous studies, a closed-sphere packing method was adopted for numerical reconstruction in this work. First, continuous or semi-continuous thin shell-like voxels were generated on the surfaces of carbon particles to represent the coating effect after heat treatment. Subsequently, elongated chain-like voxels were formed in the contact regions between adjacent carbon particles to simulate the bridging effect associated with interparticle adhesion [22]. During the implementation, the minimum distance *d_b_* (where the subscript *b* indicates the carbon sphere being considered) from each pore voxel to the nearest carbon sphere surface was calculated, and a critical radius *r_b_* was defined. When the condition *d_b_* ≥ *r_b_* was satisfied, the region was retained as a gas transport pathway; otherwise, the region was filled with PTFE. The PTFE generation process was described by the following equation:

(4) $$\varepsilon_{p}=\frac{V_{\mathrm{PTFE}}}{V_{\mathrm{PTFE}}+V_{C}}$$

where *ε_p_* represents the target PTFE content, $V_{\mathrm{PTFE}}$ is the volume of PTFE, and *V_C_* is the volume of carbon spheres.

By adjusting *r_b_* and *ε_p_*, the spatial distribution density of PTFE can be effectively controlled, thereby reproducing the hydrophobization characteristics under different processing conditions [23]. Both microstructural reconstruction and experimental observations indicate that PTFE tends to preferentially accumulate on the surfaces of carbon agglomerates. This approach enables simultaneous representation of the coating effect of PTFE on carbon particles and its bridging effect between adjacent particles. Furthermore, through parametric adjustments, various spatial distribution patterns can be constructed, providing structured inputs for subsequent transport property studies.

### 2.2. Random Walk Algorithm

Upon completion of the MPL structural reconstruction, a random walk algorithm was employed to simulate the diffusion behavior of species within the porous MPL structure. By simulating the random motion of particles in space, this algorithm enables effective calculation of transport properties such as tortuosity and effective diffusivity. After obtaining the three-dimensional reconstructed structure, equivalent transport parameters, including tortuosity, gas permeability, and effective diffusion coefficient, were extracted [24]. The random walk method, a particle tracking technique based on statistical physics, can be directly applied to voxelated pore structures to compute the macroscopic transport properties of complex porous media [25].

During the simulation, a large number of virtual particles were released into the pore space and advanced stepwise according to Brownian motion. The mean square displacement (MSD) was calculated as follows:

(5) $$r^{2}(t)=\frac{1}{n}\sum_{i=1}^{n}\left[{\left(x_{i}(t)-x_{i}(0)\right)}^{2}+{\left(y_{i}(t)-y_{i}(0)\right)}^{2}+{\left(z_{i}(t)-z_{i}(0)\right)}^{2}\right]$$

According to the linear relationship between the mean square displacement and time, the tortuosity can be defined by the following relation:

(6) $$\tau =\frac{\frac{d\langle r^{2}(t)\rangle }{dt}{|}_{free}}{\frac{d\langle r^{2}(t)\rangle }{dt}{|}_{pore}}$$

where $\frac{d\langle r^{2}(t)\rangle }{dt}{|}_{free}$ represents the rate of change in the mean square displacement of particles in free space, and $\frac{d\langle r^{2}(t)\rangle }{dt}{|}_{pore}$ represents that in the pore space. The ratio of these rates defines the tortuosity *τ*.

For flow problems, the permeability K can be obtained using the following equation [17]:

(7) $$K=\frac{\varepsilon p^{2}}{6\pi (S/V)}$$

where *ε* is the porosity, p is the characteristic pore diameter, and S/V is the specific surface area.

The effective diffusion coefficient is given by [17]:

(8) $$D_{\mathrm{eff}}=\frac{\varepsilon }{6\Delta t}{\left(\frac{dr^{2}}{dt}\right)}_{p}$$

where *ε* is the porosity, Δ*t* is the time step, and the derivative term in parentheses is calculated from the particle trajectories within the pore space.

## 3. Results and Discussion

### 3.1. Effect of Porosity

**Figure 1:**
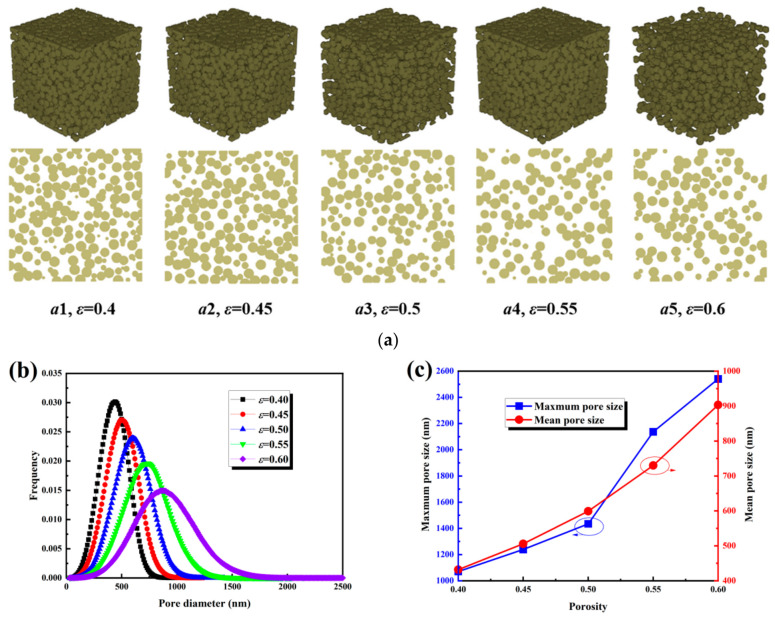
Structural characteristics of the MPL under different porosity conditions: (**a**) three-dimensional structures and cross-sectional views; (**b**) pore size distributions; (**c**) maximum and mean pore sizes.

Porosity is a key parameter governing the microstructure and transport behavior of the MPL. Based on the stochastic reconstruction model described in Section 2.1, 1000 MPL samples were generated within a porosity range of 0.40–0.60, with a sample size of 5 × 5 × 5 µm^3^ and a carbon sphere radius of 400 nm. As shown in Figure 1a, with increasing porosity, the pore space expanded, the distribution of carbon spheres became sparser, and the pore size distribution broadened significantly. Figure 1b further indicates that the pore size distribution shifted and broadened toward larger pore sizes, with the main peak shifting monotonically to the right as porosity increased. At a porosity of 0.40, the main peak was located at 450–500 nm; at porosities of 0.45, 0.50, and 0.55, it increased to 550 nm, 650 nm, and 750–800 nm, respectively; and at 0.60, it further shifted to 900–1000 nm. Meanwhile, the peak frequency decreased from 0.030 to 0.015, indicating a transition from a narrow, concentrated distribution to a broad dispersed distribution, with a pronounced enhancement of the large-pore tail, which extended beyond 2000 nm at a porosity of 0.60. As illustrated in Figure 1c, the pore size exhibited a nonlinear increase with porosity. The maximum pore size increased from 1100 nm to 1250 nm at 0.45 and to 1450 nm at 0.50, then increased abruptly to 2150 nm between 0.50 and 0.55, reaching 2550 nm at 0.60. The average pore size increased sequentially from 440 nm to 520 nm, 600 nm, and 740 nm, reaching 900–930 nm at a porosity of 0.60.

This non-linear growth and broadening of the pore size distribution can be physically explained by the geometric constraints and partial overlap of carbon spheres during the random packing process. At lower porosities, the dense packing limits the formation of large voids, resulting in relatively modest pore size growth. As the porosity increases, reduced packing density allows neighboring voids to merge and coalesce, producing abrupt increases in the largest pores and a pronounced tail in the distribution. This coalescence enhances connectivity and creates more tortuous yet interconnected pathways, directly influencing gas diffusion and liquid water transport within the MPL.

**Figure 2:**
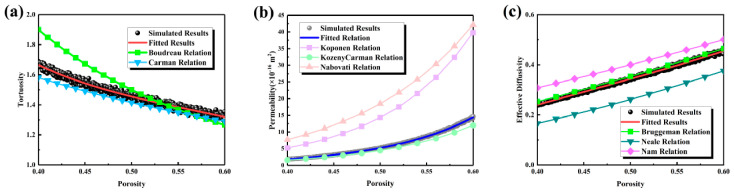
Transport properties of the MPL under different porosity conditions: (**a**) tortuosity; (**b**) permeability; (**c**) effective diffusivity.

The transport characteristics are presented in Figure 2. As shown in Figure 2a, tortuosity decreased monotonically with increasing porosity, from approximately 1.7 at a porosity of 0.40 to about 1.3 at 0.60, indicating a significant simplification of the transport pathways. As can be seen from Figure 2b, permeability increased significantly from 2 × 10^−16^ m^2^ to 13 × 10^−16^ m^2^, reflecting the enhancement of interconnected pore channels. From Figure 2c, the effective diffusivity was observed to increase from 0.25 to 0.45. Compared with classical empirical relationships shown in Table 1, the Boudreau and Carman relations overestimated and underestimated tortuosity, respectively. The Kozeny–Carman relationship captured the trend of permeability but exhibited systematic deviations, while the Bruggeman relationship showed a similar trend for effective diffusivity but failed to capture the subtle curvature of the scattered data. Equations (9)–(11), derived from fitting the simulation data, demonstrated higher accuracy. In summary, porosity effectively reduces tortuosity and simultaneously enhances permeability and effective diffusivity by enlarging pore sizes and broadening the pore size distribution.

(9) $$\tau =-150.606\varepsilon^{2}+23.099\varepsilon -7.016\ln(\varepsilon )-12.305,\;R^{2}=0.9790$$

(10) $$K=484.090\varepsilon^{7.35}+4.430\ln(\varepsilon )+5.310,\;R^{2}=0.9996$$

(11) $$D_{eff}=2.238\varepsilon^{3}-2.983\varepsilon^{2}+2.355\varepsilon -0.368,\;R^{2}=0.9970$$

### 3.2. Effect of Carbon Sphere Radius

**Figure 3:**
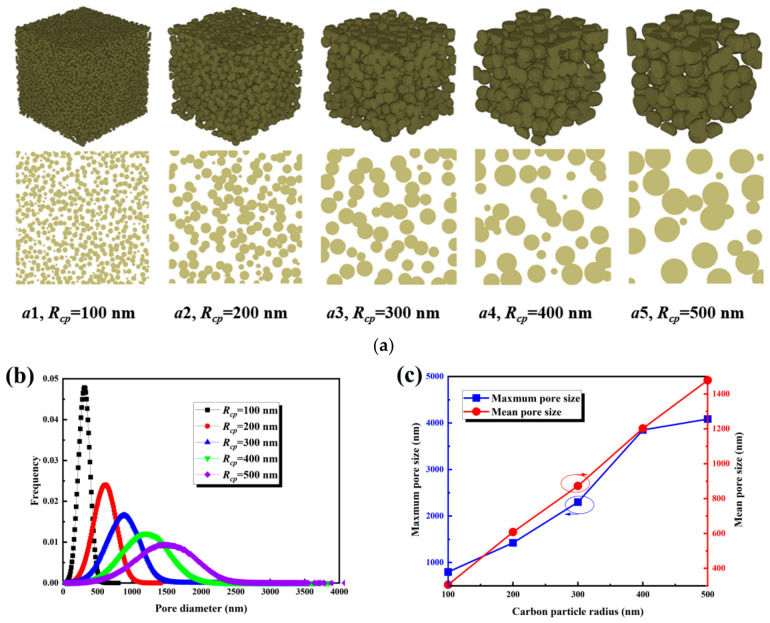
Structural characteristics of the MPL under different carbon sphere radius: (**a**) three-dimensional structures and cross-sectional views; (**b**) pore size distributions; (**c**) maximum and mean pore sizes.

The carbon sphere radius is a key structural parameter that determines the pore size and connectivity of the MPL. As shown in Figure 3a, increasing the carbon sphere radius from 100 nm to 500 nm transforms the packing of carbon spheres from dense to loose, enlarges the pore space, and makes the overall structure more open. The pore size distributions under different carbon sphere radii are presented in Figure 3b. As the radius increases, the distribution broadens significantly, indicating that larger spheres not only increase the average pore size but also enhance heterogeneity in the pore network. As illustrated in Figure 3c, the average pore size increases progressively from approximately 300 nm at 100 nm radius to 1500 nm at 500 nm, showing a nearly linear trend. The broadening of the distribution with increasing sphere radius reflects the emergence of larger voids and a more interconnected network, which provides more continuous pathways for gas transport. This evolution of pore structure due to increasing carbon sphere radius reduces the tortuosity of the transport pathways, enhances permeability, and improves effective diffusivity. The combination of larger average pore size and increased connectivity illustrates why carbon sphere radius is a dominant parameter in regulating MPL transport properties.

The observed decrease in tortuosity with increasing carbon sphere radius can be explained by the formation of larger, more interconnected pores that create more direct pathways for gas transport. Larger spheres reduce the number of constrictions and bottlenecks, simplifying the network of transport channels. However, excessively large radii could compromise mechanical support of the MPL and negatively impact water management, suggesting that an optimal radius likely exists where transport efficiency is maximized without detrimental effects on structural integrity.

**Figure 4:**
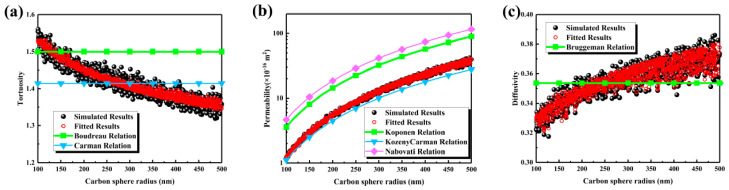
Transport properties of the MPL under different carbon sphere radius: (**a**) tortuosity; (**b**) permeability; (**c**) effective diffusivity.

The transport characteristics are presented in Figure 4. As shown in Figure 4a, tortuosity decreased from 1.55 to 1.35 as the radius increased from 100 nm to 500 nm, indicating a progressive simplification of transport pathways. In contrast to the Boudreau and Carman empirical relationships, significant deviations were observed; therefore, a fitting Equation (12) was proposed based on the simulation data. As shown in Figure 4b, permeability increased significantly from 2 × 10^−16^ m^2^ to 20 × 10^−16^ m^2^ demonstrating that the enlarged pore scale effectively enhanced through-plane flow capability. While the Kozeny–Carman, Koponen, and Nabovati correlations captured the increasing trend, discrepancies were noted; Equation (13) showed better agreement with the simulation data. As illustrated in Figure 4c, effective diffusivity gradually increased from 0.32 to 0.36, suggesting that enhanced pore connectivity and simplified pathways jointly improved diffusion efficiency. The Bruggeman relationship exhibited minor deviations, prompting the development of Equation (14) to improve prediction accuracy. In summary, increasing the carbon sphere radius shifts the pore size distribution to the right and broadens it, substantially increases both the maximum and average pore sizes, and concurrently reduces tortuosity while enhancing permeability and effective diffusivity. These combined effects lead to improved mass transport conditions from both structural and transport perspectives.

The deviations observed between classical empirical models and the simulation results can be attributed to the assumptions underlying these models. Many models, such as Boudreau, Carman, and Bruggeman, assume isotropic and homogeneously distributed pores, which neglects the heterogeneity and anisotropy introduced by random packing of carbon spheres in the reconstructed MPL. This results in systematic under- or overestimation of tortuosity, permeability, and diffusivity, particularly at larger sphere radii where the pore network becomes more heterogeneous. Including metrics such as RMSE or relative error highlights these discrepancies quantitatively, confirming that empirical models are limited in capturing the subtle structural effects observed in the simulations.

(12) $$\tau =\left(-150.606\varepsilon^{2}+23.099\varepsilon -7.016\ln(\varepsilon )-12.305\right)\cdot 1.233\cdot d^{-0.067}\cdot \exp\left(-3.20\times 10^{-4}\cdot d\right),R^{2}=0.9121$$

(13) $$K=\left(484.090\varepsilon^{7.35}+4.430\ln(\varepsilon )+5.310\right)\cdot \left(9.10\times 10^{-4}d^{2.284}+0.141\ln(d)-0.269\right),R^{2}=0.9983$$

(14) $$D_{eff}=\left(2.238\varepsilon^{3}-2.983\varepsilon^{2}+2.355\varepsilon -0.368\right)\cdot 0.838\cdot d^{0.053}\cdot \exp\left(8.56\times 10^{-4}\cdot d\right),R^{2}=0.9203$$

### 3.3. Effect of Maximum Overlap Ratio

**Figure 5:**
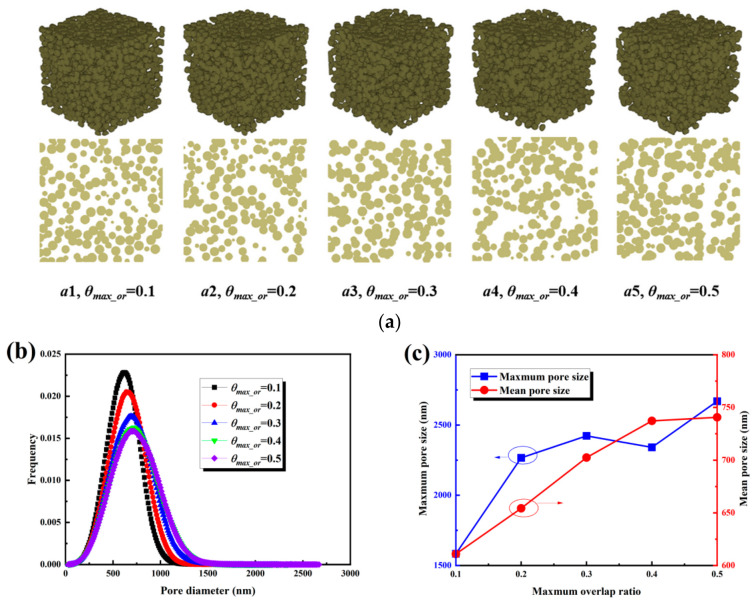
Structural characteristics of the MPL under different maximum overlap ratios: (**a**) three-dimensional structures and cross-sectional views; (**b**) pore size distributions; (**c**) maximum and mean pore sizes.

The maximum overlap ratio is an important parameter affecting the pore structure of the MPL. As shown in Figure 5a, with an increase in the maximum overlap ratio, the geometric overlap between carbon spheres intensified, the agglomeration of the solid phase became more pronounced, and the pore morphology became more irregular. It should be noted that while the overall porosity did not necessarily change significantly, the pore size distribution shifted toward larger scales. The variation in pore size distribution with the maximum overlap ratio is presented in Figure 5b. The main peak shifted monotonically to the right as the ratio increased: as the ratio increased from 0.1 to 0.5, the peak value increased sequentially from 600 nm to 650, 700, 750, and 800 nm, accompanied by an enhancement of the large-pore tail and a significant broadening of the distribution range. The evolution of characteristic pore sizes is illustrated in Figure 5c. When the ratio increased from 0.1 to 0.3, the maximum pore size increased from 1600 nm to 2400 nm, and the average pore size increased from 610 nm to 700 nm. At a ratio of 0.4, the maximum pore size decreased to 2300 nm, while the average pore size increased to 735 nm. At a ratio of 0.5, the maximum pore size rebounded to 2600 nm, and the average pore size reached 755 nm. The maximum pore size increased by approximately 1000 nm, significantly larger than the increase of about 145 nm observed for the average pore size.

**Figure 6:**
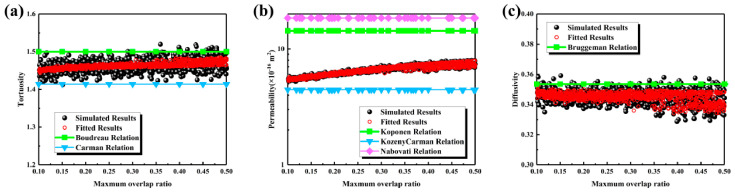
Transport properties of the MPL under different maximum overlap ratios: (**a**) tortuosity; (**b**) permeability; (**c**) effective diffusivity.

The transport characteristics are presented in Figure 6. As shown in Figure 6a, tortuosity remained stable within the range of 1.4 to 1.5 as the maximum overlap ratio increased from 0.1 to 0.5. The Boudreau relationship overestimated tortuosity, while the Carman relationship underestimated it; therefore, a fitting Equation (15) was proposed based on the simulation data. As shown in Figure 6b, permeability increased slowly from 6 × 10^−16^ m^2^ to 8 × 10^−16^ m^2^, corresponding to an increase of approximately 33%. Existing empirical relationships failed to accurately capture the bandwidth of the scattered data, whereas Equation (16) exhibited higher accuracy. As illustrated in Figure 6c, effective diffusivity fluctuated within a narrow range of 0.34 to 0.36. The Bruggeman relationship showed deviations, and Equation (17) provided a better fit to the simulation data. In summary, the maximum overlap ratio primarily enhances permeability to a moderate extent by shifting the main peak of the pore size distribution and significantly increasing the maximum pore size, while its influence on tortuosity and effective diffusivity is limited.

(15) $$\tau =\left(-150.606\varepsilon^{2}+23.099\varepsilon -7.016\ln(\varepsilon )-12.305\right)\cdot \left(0.224d^{3}-0.285d^{2}+0.142d+0.989\right),R^{2}=0.7286$$

(16) $$K=\left(484.090\varepsilon^{7.35}+4.430\ln(\varepsilon )+5.310\right)\cdot \left(0.866+1.489d-1.223d^{2}+0.594d^{3}\right),R^{2}=0.9860$$

(17) $$D_{eff}=\left(2.238\varepsilon^{3}-2.983\varepsilon^{2}+2.355\varepsilon -0.368\right)\cdot 0.971\cdot d^{-0.012}\cdot \exp\left(9.15\times 10^{-3}\cdot d\right),R^{2}=0.7124$$

### 3.4. Effect of Seed Ratio

**Figure 7:**
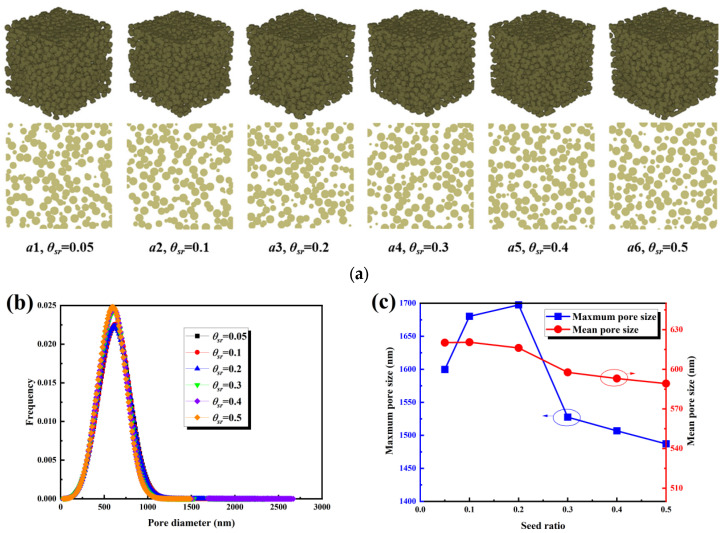
Structural characteristics of the MPL under different seed ratios: (**a**) three-dimensional structures and cross-sectional views; (**b**) pore size distributions; (**c**) maximum and mean pore sizes.

The seed ratio is an important parameter for regulating the pore structure of the MPL. As shown in Figure 7a, with an increase in the seed ratio, the spatial distribution of initial nucleation sites changed, the aggregation morphology of carbon spheres was altered, and the pore network transitioned from a relatively uniform structure to one characterized by localized agglomeration and pore reconstruction. The variation in pore size distribution with the seed ratio is presented in Figure 7b. At a seed ratio of 0.05, the main peak was located at approximately 500 nm with a narrow distribution. At 0.10, the main peak shifted to 600 nm with a noticeable broadening. When the seed ratio reached 0.20, the distribution no longer continued to shift to the right, exhibiting a trend of initial expansion followed by contraction. As illustrated in Figure 7c, the characteristic pore sizes exhibited a non-monotonic evolution. As the seed ratio increased from 0.05 to 0.20, the maximum pore size increased from 1600 nm to 1700 nm, while the average pore size remained around 610 nm. When the ratio increased to 0.30, the maximum pore size decreased sharply to 1525 nm, and the average pore size dropped to 590 nm. At a ratio of 0.50, the maximum pore size further decreased to 1475 nm, and the average pore size decreased to 575 nm. The simultaneous decline in both maximum and average pore sizes after a ratio of 0.20 indicates that a high seed ratio induces localized stacking and pore throat refinement, leading to the fragmentation of large pores.

**Figure 8:**
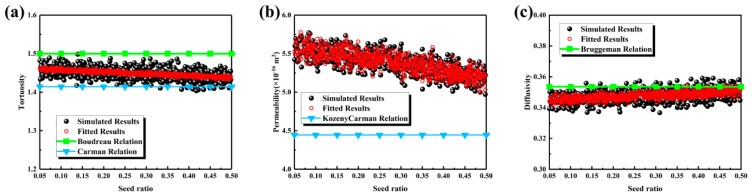
Transport properties of the MPL under different seed ratios: (**a**) tortuosity; (**b**) permeability; (**c**) effective diffusivity.

The transport characteristics are presented in Figure 8. As shown in Figure 8a, tortuosity remained stable within the range of 1.4 to 1.5 as the seed ratio increased from 0.05 to 0.50, indicating limited variation in the degree of pathway tortuosity. Systematic deviations were observed in the Boudreau and Carman relationships; therefore, a fitting Equation (18) was proposed based on the simulation data. As shown in Figure 8b, permeability exhibited a slight overall decrease, maintaining approximately 5.5 × 10^−16^ m^2^ when the ratio increased from 0.05 to 0.20, and decreasing to 5.2 × 10^−16^ m^2^ when the ratio increased to 0.50, consistent with the decreasing trend in characteristic pore sizes. The Kozeny–Carman relationship slightly underestimated permeability, whereas Equation (19) provided a better fit to the simulation data. As illustrated in Figure 8c, effective diffusivity increased slightly from 0.34 to 0.35, indicating that diffusion was primarily governed by pore connectivity and volume fraction. Although the Bruggeman relationship captured the general trend, deviations were observed; Equation (20) improved prediction accuracy. In summary, the effect of seed ratio on the microstructure exhibited a non-monotonic behavior, with a larger characteristic pore size observed at a ratio of approximately 0.20. Higher ratios led to pore throat refinement and a mild reduction in permeability, while their influence on tortuosity and effective diffusivity was limited.

(18) $$\tau =\left(-150.606\varepsilon^{2}+23.099\varepsilon -7.016\ln(\varepsilon )-12.305\right)\cdot \left(1.009-0.032d-0.004d^{2}\right),R^{2}=0.7494$$

(19) $$K=\left(484.090\varepsilon^{7.35}+4.430\ln(\varepsilon )+5.310\right)\cdot \left(-0.092d^{3}-0.038d^{2}+0.129d+1.039\right),R^{2}=0.8723$$

(20) $$D_{eff}=\left(2.238\varepsilon^{3}-2.983\varepsilon^{2}+2.355\varepsilon -0.368\right)\cdot \left(0.992+0.030d+0.007d^{2}\right),R^{2}=0.7271$$

### 3.5. Effect of PTFE Content

**Figure 9:**
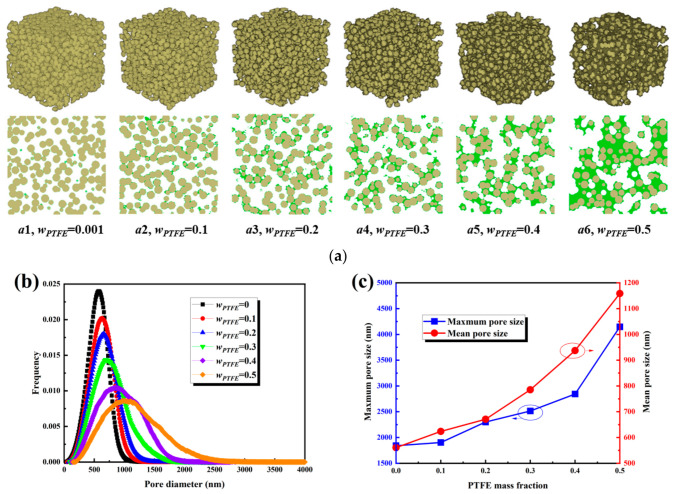
Structural characteristics of the MPL under different PTFE contents: (**a**) three-dimensional structures and cross-sectional views; (**b**) pore size distributions; (**c**) maximum and mean pore sizes.

PTFE content is a critical factor influencing the pore structure and transport properties of the MPL. As shown in Figure 9a, with increasing PTFE content from 0 to 0.5, the gaps between carbon spheres enlarged, the pore space expanded significantly, and the structure became more open. The variation in pore size distribution with PTFE content is presented in Figure 9b. The main peak shifted monotonically to the right and broadened progressively: at a PTFE content of 0, the peak was approximately 600 nm; at contents of 0.1, 0.2, and 0.3, it increased to 650, 700, and 750 nm, respectively; at 0.4, it shifted to 900 nm; and at 0.5, it reached 1000 nm. As illustrated in Figure 9c, the characteristic pore sizes increased correspondingly: at a PTFE content of 0, the maximum and average pore sizes were 1575 nm and 550 nm, respectively; at 0.1, they were 1590 nm and 650 nm; at 0.2, they increased to 2250 nm and 675 nm; at 0.3, they increased to 2500 nm and 800 nm; at 0.4, they reached 2575 nm and 950 nm; and at 0.5, they increased significantly to 4500 nm and 1150 nm. The maximum pore size exhibited a stepwise increase after a PTFE content of 0.2, while the average pore size increased more rapidly in the range of 0.3 to 0.5.

**Figure 10:**
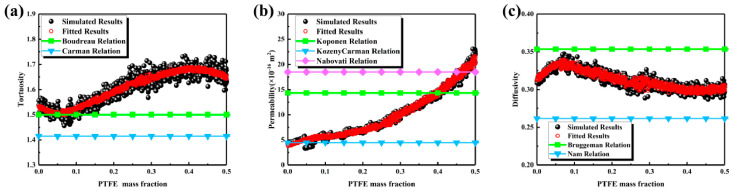
Transport properties of the MPL under different PTFE contents: (**a**) tortuosity; (**b**) permeability; (**c**) effective diffusivity.

The transport characteristics are presented in Figure 10. As shown in Figure 10a, tortuosity increased gradually with increasing PTFE content, rising from approximately 1.5 at a PTFE content of 0 to 1.65 at 0.5. Empirical relationships were relatively accurate at low PTFE contents but underestimated tortuosity at high PTFE contents; therefore, Equation (21) was proposed to compensate for this systematic deviation. As shown in Figure 10b, permeability was most sensitive to PTFE content, increasing from 5 × 10^−16^ m^2^ at a PTFE content of 0 to 12 × 10^−16^ m^2^ at 0.3, and reaching 22.5 × 10^−16^ m^2^ at 0.5, approximately 4.5 times the initial value. The Kozeny–Carman relationship was relatively accurate at low PTFE contents but exhibited increasing deviation at high PTFE contents; Equation (22) provided more stable predictions. As illustrated in Figure 10c, effective diffusivity exhibited a non-monotonic trend, increasing from 0.31 at a PTFE content of 0 to 0.33 at 0.1, then gradually decreasing to 0.31 at 0.5. The Bruggeman and Nam relationships were unable to capture the details of the initial increase followed by a decrease; Equation (23) was found to be more suitable for the present system. In summary, PTFE content significantly enhanced permeability by coarsening the pore structure, while tortuosity increased slowly and effective diffusivity exhibited a non-monotonic trend.

Although PTFE is hydrophobic and can partially block pores, in the reconstructed MPL models its addition primarily increases the spacing between carbon spheres, effectively enlarging the pore throats and coalescing smaller voids. This coarsening of the pore network dominates over the local blocking effect of PTFE, resulting in a net increase in permeability. At low PTFE contents, the improvement is modest because the network is still constrained by carbon packing, whereas at higher PTFE contents, the pore network becomes more open and interconnected, enhancing through-plane flow. This explains the counterintuitive trend of increasing permeability despite the hydrophobic nature of PTFE.

Further clarification of the physical mechanism: In the present reconstruction framework, PTFE is not modeled as a phase that redistributes or replaces carbon particles; instead, it is introduced as a secondary phase that modifies the local geometry by coating carbon surfaces and forming bridge-like connections between adjacent particles, consistent with experimentally observed PTFE distributions in MPLs [22,23]. This treatment effectively alters pore morphology rather than displacing the solid carbon framework. As a result, PTFE addition induces both pore throat enlargement and local pore coalescence, especially at higher contents where adjacent voids merge due to reduced geometric confinement. Similar behavior has been reported in experimental and pore-scale simulation studies, where PTFE redistribution leads to restructuring of pore networks and modification of transport pathways [18,22]. Therefore, the increase in permeability can be understood as a competition between local pore blockage and global structural reorganization, with the latter dominating under the present parameter range.

(21) $$\tau =\left(-10.606\varepsilon^{2}+23.099\varepsilon -7.016\ln(\varepsilon )-12.305\right)\cdot \left(1.029-6.0\times 10^{-4}d+2.440d^{2}-4.073d^{3}\right),R^{2}=0.9161$$

(22) $$K=\left(484.090\varepsilon^{7.35}+4.430\ln(\varepsilon )+5.310\right)\cdot \left(0.901+1.510d-2.416d^{2}+24.443d^{3}\right),R^{2}=0.9869$$

(23) $$D_{eff}=\left(2.238\varepsilon^{3}-2.983\varepsilon^{2}+2.355\varepsilon -0.368\right)\cdot \left(0.973-0.051d-1.831d^{2}+3.172d^{3}\right),R^{2}=0.9119$$

## 4. Conclusions

Based on the above investigations, a three-dimensional microstructural model of the MPL with tunable structural parameters was constructed using a stochastic reconstruction method. Combined with random walk algorithms and pore-scale transport calculations, the effects of key structural parameters—including porosity, carbon sphere radius, maximum overlap ratio, seed ratio, and PTFE content—on permeability, oxygen effective diffusivity, and tortuosity were systematically evaluated.

From a physical perspective, the transport behavior of the MPL is governed by the interplay between pore size distribution, pore connectivity, and geometric constraints imposed by carbon sphere packing and PTFE distribution. In general, transport properties are controlled by pore enlargement and coalescence, pathway connectivity, and the competition between pore opening and local blockage effects.

The main conclusions are summarized as follows:

Increasing porosity significantly enlarges pore size and broadens its distribution, leading to reduced tortuosity and enhanced permeability and effective diffusivity. Increasing carbon sphere radius coarsens the pore structure and improves connectivity, resulting in lower tortuosity and higher transport performance. The maximum overlap ratio mainly enlarges extreme pore sizes and moderately improves permeability, with limited influence on tortuosity and diffusivity. The seed ratio exhibits a non-monotonic effect, with an optimal value (~0.20) that balances pore enlargement and structural uniformity. PTFE content strongly affects permeability by modifying pore geometry; while it enhances flow transport through pore coarsening, excessive PTFE increases tortuosity and reduces diffusion efficiency.

High-precision fitting equations were proposed to describe the relationships between structural parameters and transport properties, providing a quantitative basis for MPL design. The results indicate that porosity and carbon sphere radius are the dominant factors, while PTFE content plays a key role in regulating permeability.

Future work will focus on coupling multiphase transport processes, incorporating experimental validation, and exploring multi-parameter optimization strategies to achieve a balance between transport performance, mechanical stability, and water management.

## Figures and Tables

**Table 1:** The predictive correlation relation for transport parameters proposed in previous studies.

Author	Parameter	Relation
Boudreau et al. [26]	Tortuosity	$\tau =1+\frac{{\left(1-\varepsilon \right)}^{2}}{\varepsilon }$
Carman [27]	Tortuosity	$\tau =\varepsilon^{-0.5}$
Koponen et al. [28]	Permeability	$K=\frac{5.55r^{2}}{\exp\left[10.1(1-\varepsilon )\right]-1}$
Kozeny–Carman	Permeability	$K=\frac{d^{2}}{180}\cdot \frac{\varepsilon^{3}}{{\left(1-\varepsilon \right)}^{2}}$
Nabovati et al. [29]	Permeability	$K=0.491r^{2}{\left(\sqrt{\frac{0.9257}{1-\varepsilon }}-1\right)}^{2.31}$
Bruggeman et al. [30]	Effective diffusivity	$D_{\mathrm{eff}}=\varepsilon^{1.5}$
Neale et al. [31]	Effective diffusivity	$D_{\mathrm{eff}}=\frac{2\varepsilon }{3-\varepsilon }$
Nam et al. [32]	Effective diffusivity	$D_{\mathrm{eff}}=\varepsilon {\left(\frac{\varepsilon -0.11}{0.89}\right)}^{0.785}$

## Data Availability

The original contributions presented in this study are included in the article. Further inquiries can be directed to the corresponding authors.

## References

[1] Dai J., Gao Y., Zhang T. (2023). Analysis on the Influence of Crack Structure on MPL Transmission Properties. J. Power Sources.

[2] Qian Z., Fan Y., Yue L., Zhu Y., Wang S., Miyazawa A., Ozaki S. (2023). Performance of Proton Exchange Membrane Fuel Cells with Microporous Layer Hydrophobized by Polyphenylene Sulfide at Conventional Temperature and Cold Start. Int. J. Hydrogen Energy.

[3] Orogbemi O.M., Ingham D.B., Ismail M.S., Hughes K.J., Ma L., Pourkashanian M. (2018). On the Gas Permeability of the Microporous Layer Used in Polymer Electrolyte Fuel Cells. J. Energy Inst..

[4] Son J., Um S., Kim Y.-B. (2021). Numerical Analysis of the Effect of Anisotropic Gas Diffusion Layer Permeability on Polymer Electrolyte Membrane Fuel Cell Performance with Various Channel Types. Fuel.

[5] Lee F.C., Ismail M.S., Zhang K., Ingham D.B., Aldakheel F., Hughes K.J., Ma L., El-Kharouf A., Pourkashanian M. (2023). Optimisation and Characterisation of Graphene-Based Microporous Layers for Polymer Electrolyte Membrane Fuel Cells. Int. J. Hydrogen Energy.

[6] Kim J., Kim H., Song H., Kim D., Kim G.H., Im D., Jeong Y., Park T. (2021). Carbon Nanotube Sheet as a Microporous Layer for Proton Exchange Membrane Fuel Cells. Energy.

[7] Zhang H., Zhu L., Sarker M., Wang M., Chang H., Kui D., Zhan Z. (2025). Pore-Scale Simulations Investigating Mechanical Compression Effects on Transport Properties at Gas Diffusion Layer/Micro-Porous Layer Interfaces in Proton Exchange Membrane Fuel Cells. J. Power Sources.

[8] Ye S., Hou Y., Li X., Jiao K., Du Q. (2022). Pore-Scale Investigation of Coupled Two-Phase and Reactive Transport in the Cathode Electrode of Proton Exchange Membrane Fuel Cells. Trans. Tianjin Univ..

[9] Nouri-Khorasani A., Bonakdarpour A., Fang B., Wilkinson D.P. (2022). Rational Design of Multimodal Porous Carbon for the Interfacial Microporous Layer of Fuel Cell Oxygen Electrodes. ACS Appl. Mater. Interfaces.

[10] Lee J., Banerjee R., George M.G., Muirhead D., Shrestha P., Liu H., Ge N., Chevalier S., Bazylak A. (2017). Multiwall Carbon Nanotube-Based Microporous Layers for Polymer Electrolyte Membrane Fuel Cells. J. Electrochem. Soc..

[11] Ren G., Qu Z., Hai Y., Wang Y. (2024). Liquid and Gas Permeabilities of Nanostructured Layers: Three-Dimensional Lattice Boltzmann Simulation. Int. J. Hydrogen Energy.

[12] Ma J., Zhang X., Jiang Z., Ostadi H., Jiang K., Chen R. (2014). Flow Properties of an Intact MPL from Nano-Tomography and Pore Network Modelling. Fuel.

[13] Zhang H., Shao X., Zhan Z., Sarker M., Sui P.-C., Chuang P.-Y.A., Pan M. (2023). Pore-Scale Modeling of Microporous Layer for Proton Exchange Membrane Fuel Cell: Effective Transport Properties. Membranes.

[14] Uenishi T., Imoto R. (2023). Experimental and Numerical Analysis of a Catalyst Layer in the Membrane Electrode Assembly of Polymer Electrolyte Membrane Fuel Cells. J. Power Sources.

[15] Wang H., Li L., Yang G., Huang N. (2025). Nanoscale Simulation of Ice Melting Behavior and Thermal Conductivity Characteristics of Catalyst Layers in Proton Exchange Membrane Fuel Cells Using the Lattice Boltzmann Method. Fuel.

[16] Liu Q., Lan F., Wang J., Chen J., Zeng C. (2022). Numerical Effect of Random Poral Microstructures in Stacking Gas Diffusion Layers on Water Transport Capability. J. Power Sources.

[17] Jia Q., Wang H., Yang G. (2025). Structural Parameters on the Effective Transport Properties of Carbon Cloth Gas Diffusion Layers: Random Walk Simulations. Nanomaterials.

[18] Orogbemi O.M., Ingham D.B., Ismail M.S., Hughes K.J., Ma L., Pourkashanian M. (2016). The Effects of the Composition of Microporous Layers on the Permeability of Gas Diffusion Layers Used in Polymer Electrolyte Fuel Cells. Int. J. Hydrogen Energy.

[19] Sim J., Kang M., Min K. (2021). Effects of Basic Gas Diffusion Layer Components on PEMFC Performance with Capillary Pressure Gradient. Int. J. Hydrogen Energy.

[20] He Y., Hao L., Bai M. (2024). Pore-Scale Simulation of Tortuosity in the Catalyst Layer of Proton Exchange Membrane Fuel Cells. J. Energy Eng..

[21] Gao Y., Jin T., Wu X. (2020). Stochastic 3D Carbon Cloth GDL Reconstruction and Transport Prediction. Energies.

[22] Van Truong M., Wang C.-L., Yang M., Yang H. (2018). Effect of Tunable Hydrophobic Level in the Gas Diffusion Substrate and Microporous Layer on Anion Exchange Membrane Fuel Cells. J. Power Sources.

[23] Yoshimune W., Harada M., Akimoto Y. (2020). Small-Angle Neutron Scattering Studies on the Distribution of Polytetrafluoroethylene within Microporous Layers for Polymer Electrolyte Fuel Cells. Compos. Part C Open Access.

[24] Huang J., Xiao F., Dong H., Yin X. (2019). Diffusion Tortuosity in Complex Porous Media from Pore-Scale Numerical Simulations. Comput. Fluids.

[25] Jia Q., Wang H., Yang G. (2025). Pore-Scale Analysis of Structural Properties and Transfer Characteristics of Microporous Layers in Proton Exchange Membrane Fuel Cells. Proc. SPIE.

[26] Boudreau B.P., Meysman F.J.R. (2006). Predicted Tortuosity of Muds. Geology.

[27] Carman P.C. (1997). Fluid Flow through Granular Beds. Chem. Eng. Res. Des..

[28] Koponen A., Kandhai D., Hellén E., Alava M., Hoekstra A., Kataja M., Niskanen K., Sloot P., Timonen J. (1998). Permeability of Three-Dimensional Random Fiber Webs. Phys. Rev. Lett..

[29] Nabovati A., Llewellin E.W., Sousa A.C.M. (2009). A General Model for the Permeability of Fibrous Porous Media Based on Fluid Flow Simulations Using the Lattice Boltzmann Method. Compos. Part A Appl. Sci. Manuf..

[30] Bruggeman D.A.G. (1935). Berechnung Verschiedener Physikalischer Konstanten von Heterogenen Substanzen. I. Dielektrizitätskonstanten Und Leitfähigkeiten Der Mischkörper Aus Isotropen Substanzen. Ann. Phys..

[31] Neale G.H., Nader W.K. (1973). Prediction of Transport Processes within Porous Media: Diffusive Flow Processes within an Homogeneous Swarm of Spherical Particles. AIChE J..

[32] Nam J.H., Kaviany M. (2003). Effective Diffusivity and Water-Saturation Distribution in Single- and Two-Layer PEMFC Diffusion Medium. Int. J. Heat Mass Transf..

